# P2X7 receptor: a potential target for treating comorbid anxiety and depression

**DOI:** 10.1007/s11302-024-10007-0

**Published:** 2024-04-20

**Authors:** Jun Liu, Ting-Ting Liu, Lan Mou, Yuwen Zhang, Xiang Chen, Qi Wang, Bin-Lu Deng, Jie Liu

**Affiliations:** 1 Department of Neurology, School of Clinical Medicine, Southwest Medical University, Luzhou, China; 2 Department of Geriatric Neurology, Qinglongchang Ward, Chengdu Sixth People’s Hospital, Chengdu, China; 3 Department of Neurology, Sichuan Academy of Medical Sciences, Sichuan Provincial People’s Hospital, Chengdu, China; 4 Department of Neurology, Sichuan Provincial People’s Hospital, University of Electronic Science and Technology of China, Chengdu, China; 5 Sichuan Provincial Center for Mental Health, Sichuan Provincial People’s Hospital, School of Medicine, University of Electronic Science and Technology of China, Chengdu, China

**Keywords:** P2X7R, Anxiety, Depression, Neuroinflammation, Comorbidity

## Abstract

In clinical practice, depression and anxiety frequently coexist, and they are both comorbid with somatic diseases. The P2X7R is an adenosine 5’-triphosphate (ATP)-gated non-selective cation channel that is widely expressed in immune-related cells. Under conditions of stress, chronic pain, and comorbid chronic physical illness, P2X7R activation in glial cells leads to neuroinflammation. This could contribute to the development of anxiety and depression-related emotional disturbances. Previous studies have shown that the P2X7 receptor (P2X7R) plays an important role in the pathogenesis of both anxiety and depression. Thus, the P2X7R may play a role in the comorbidity of anxiety and depression. Positron emission tomography can be used to assess the degree and location of neuroinflammation by monitoring functional and expression-related changes in P2X7R, which can facilitate clinical diagnoses and guide the treatment of patients with anxiety and depression. Moreover, single nucleotide polymorphisms (SNPs) in the P2X7R gene are associated with susceptibility to different types of psychiatric disorders. Thus, evaluating the SNPs of the P2X7R gene could enable personalized mood disorder diagnoses and treatments. If the P2X7R were set as a therapeutic target, selective P2X7R antagonists may modulate P2X7R function, thereby altering the balance of intra- and extra-cellular ATP. This could have therapeutic implications for treating anxiety and depression, as well as for pain management. According to in vitro and in vivo studies, the P2X7R plays an important role in anxiety and depression. In this review, we consider the potential of the P2X7R as a therapeutic target for comorbid anxiety and depression, and discuss the potential diagnostic and therapeutic value of this receptor.

## Comorbidity of anxiety and depression in clinical practice

Depression and anxiety are common mental health disorders that can significantly impact the thoughts, mood, and physical well-being of an individual [1, 2]. These conditions collectively affect more than 350 million people worldwide, and thus impose a substantial socio-economic burden [3]. Depression manifests through a spectrum of symptoms characterized by enduring feelings of profound sadness, a pervasive sense of hopelessness, and an overarching perception of worthlessness. Clinically significant features include disturbances in appetite, alterations in sleep patterns, and a discernible depletion of energy levels [4]. In clinical practice, individuals with depression frequently experience symptoms of anxiety or have comorbid anxiety disorders [5]. Studies have found that 37.3% of patients with depression have comorbid anxiety disorders, and those with symptoms of both depression and anxiety account for 74.63%. These patients often exhibit more severe anxiety and depression symptoms, experience a longer illness duration, have an elevated risk of suicide, and generally show lower rates of clinical remission. This presents a substantial challenge for clinical diagnosis and treatment [6]. Moreover, individuals with comorbid anxiety and depression frequently experience concurrent somatic diseases, which can significantly impair their quality of life and social functioning. These comorbidities represent a substantial portion of the global disease burden [7, 8].

Anxiety and depression exhibit divergence on the timing and causative factors in the etiology, despite their overlapping clinical manifestations. Anxiety disorders are often characterized by excessive worry about future events, whereas depression is frequently linked to rumination over past events [9]. This temporal distinction suggests that the timing of adverse events plays a crucial role in determining whether an individual experiences anxiety or depression. For instance, adverse events anticipated in the future may trigger anxiety, while similar events that occurred in the past are more likely to induce depressive symptoms. Both anxiety and depression share common etiological factors such as genetics, environmental influences, stress, and early trauma. However, distinct differences exist in their causes. Anxiety is often triggered by environmental contexts and fears, such as social situations or specific phobias, and is influenced by prolonged stress and behavioral learning from specific events. Depression, on the other hand, is marked by biochemical changes like reduced serotonin and norepinephrine levels, has a stronger genetic predisposition, and is closely associated with significant life events like loss or major changes. These differences suggest that anxiety treatments might focus more on managing stress and specific fears, whereas depression treatments may target biochemical imbalances, psychological therapy, and supportive interventions [10].

Anxiety and depression often coexist, revealing complex interactions through shared biological and psychological mechanisms, thereby contributing to their comorbidity and potentially exacerbating symptoms each other. An interpersonal model of comorbidity suggests that anxiety disorders disrupt interpersonal functioning, increasing the risk for depression. This model underlines how relational dysfunction can mediate the association between anxiety disorders and later depression [11]. Anxiety and depression interact in the development of mixed anxiety/depression disorder, often leading to slower treatment response, higher medication doses, and increased suicide risk. Psychotropic drugs can have limited therapeutic effects in cases of comorbid conditions, indicating independent influences on each other [12, 13]. Additionally, the interaction between anxiety and depression is evidenced by shared underlying mechanisms, such as alterations in neurotransmitter systems, inflammatory processes, and hypothalamic-pituitary-adrenal (HPA) axis dysregulation [14, 15].

Therefore, in clinical practice, more attention should be paid to the diagnosis of the comorbidity of anxiety and depression. If patients meet the criteria for depression, and at least two of the five anxiety symptoms are added, such as “feeling nervous or anxious,” “abnormally restless,” “difficulty concentrating due to worry,” “worrying about the possibility of something terrible happening,” and “feeling that one may lose control,” they should be diagnosed with depression with anxiety [16]. Compared with patients with non-anxiety-related depression, subjects with anxiety-related depression have different neurobiological characteristics. Several studies have revealed significant differences between anxiety-related depression and non-anxiety-related depression in Hypothalamic–Pituitary -Adrenal(HPA)axis function, brain structure and functional imaging results, and inflammatory markers. Compared with non-anxiety-related depression, patients with anxiety-related depression have greater HPA axis dysfunction [17, 18]. The pathogenesis of anxiety and depression is complex. It involves multiple biological factors, such as genetics, neurotransmitter imbalance, and inflammatory response, and the interaction between psychological stress and the social environment.

Clinical diagnoses of anxiety or depression are primarily determined according to clinical symptoms and questionnaire responses. Thus, they lack objective biological markers. Additionally, the clinical application of anti-anxiety and anti-depressant medications is limited because of the associated risk of side effects, slow onset of action, poor treatment response, and issues with patient compliance. As a result, there is a notable gap between the number of people diagnosed and the number of people treated for anxiety and depression. The identification of new biomarkers and drug targets could help to address these challenges.

## P2X7R-related Neuroinflammation: key contributor to depression and anxiety, respectively

The P2X7R is an adenosine 5’-triphosphate (ATP)-gated non-selective cation channel. As a member of the purinergic signaling family, it is widely distributed in the frontal cortex and hippocampus of the central nervous system. The P2X7R is predominantly found on non-neuronal cells such as astrocytes, microglial cells, and oligodendrocytes. It serves as a sensor for purine nucleotides, including ATP, and mediates neuroinflammation [19].

Previous studies have shown that the P2X7 receptor (P2X7R) plays an important role in the pathogenesis of depression. It mainly participates in the pathogenesis of depression in three ways. First, the activation of P2X7R can trigger a series of inflammatory reactions, including the release of cytokines and the activation of immune cells. Second, P2X7R can affect the release and reuptake of various neurotransmitters, such as glutamate, Gamma-Aminobutyric Acid **(**GABA), dopamine, and serotonin. These neurotransmitters play a crucial role in regulating emotions, cognition, and motivation. Therefore, P2X7R may contribute to the onset of depression by affecting the neurotransmitter balance [20]. Third, the activation of P2X7R can affect the structure and function of synapses, including synaptic transmission, synaptic long-term potentiation, and long-term inhibition. These changes in synaptic plasticity may be related to the pathophysiological mechanisms of depression; therefore, P2X7R may affect the symptoms of depression by regulating synaptic plasticity. Fourth, the interaction between depression and the immune system is closely related. P2X7R not only expresses in the nervous system, but also plays a significant role in the immune system. Therefore, P2X7R may affect the onset of depression by regulating neuroimmune interactions [3, 21, 28].

Inflammation plays an important role in the onset of depression. A growing body of evidence suggests that purinergic signaling via the extracellular release of ATP and its accumulation at sites of injury or stress can activate the P2X7R and trigger neuroinflammation. In cases of chronic stress, purinergic signaling releases extracellular ATP that accumulates at the site of injury, leading to the upregulation of the NOD-like receptor protein 3(NLRP3)inflammasome and interleukin-1β (IL-1β) levels. This activates P2X7R expression on hippocampal microglia, resulting in neuroinflammation and oxidative stress that increases the permeability of the blood–brain barrier and disrupts the normal function of neurons and glial cells. This sequence of events leads to the development of depression [3, 20, 22, 23]. Studies have shown that all forms of stress, regardless of their duration, can lead to a decrease in P2X7R expression. Compared with acute stress, chronic stress can lead to a greater decrease in the functional threshold of P2X7R, which means that P2X7R is more easily activated under chronic stress conditions with the same stimulation. This excessive activation can lead to an increase in neuroinflammation, disrupting the balance between neurotransmitters and synaptic plasticity and ultimately leading to the onset of depression [24]. Behavioral tests in mice have demonstrated that P2X7R is involved in the development of depression-like behaviors under both acute and chronic stress conditions. In cases of intense acute stress, ATP release stimulates P2X7R activation on microglial cells and astrocytes, leading to neuroinflammation and depression-like behaviors [25]. Many researches indicate a significant connection between genetic variations in immune system genes and the development of mood and anxiety disorders. Barnes et al. provide substantial evidence through a systematic review that genetic variants including those in the IL-1β gene, contribute to the immune activation observed in individuals with depression, suggesting a genetic predisposition towards an inflammatory response [26].Multiple studies have demonstrated that rats subjected to chronic and unpredictable stressors show increased eATP levels in their hippocampal glial cells, indicating sustained activation of the P2X7R/NLRP3 axis. This leads to increased P2X7R expression in the hippocampus and amygdala, resulting in hippocampal neuroinflammation and the development of depression-like and anxiety-like behaviors in rats. The administration of P2X7R antagonists, a P2X7R knockout, or the inhibition of NLRP3 inflammasome activity can alleviate chronic stress-induced depression-like behaviors in rats [27, 28]. Further clinical research is needed to determine whether P2X7R can serve as a potential therapeutic target for human antidepressants.

Animal model studies have shown that stress leads to a massive outflow of ATP in the brain, stimulating P2X7Rs, which on their behalf, trigger the release of IL-1β. Then, IL-1β induces the secretion of a corticotropin-releasing hormone (CRH) and the consecutive production of adrenocorticotropic hormone (ACTH), resulting in mood disorders [29].

Under conditions of persistent stress, systemic levels of glucocorticoids increase, inducing the release of high-mobility group box-1 (HMGB1) from astrocytes in rats through membrane protein-1 and P2X7R. This leads to a state of neuroinflammation, which has been associated with the development of major depressive disorder [30]. Conditional humanized mice bred using different Cre transgenes can express the human P2X7R in different areas and cell types within the central nervous system. Overactivation of the P2X7R can lead to increased neuronal excitability and anxiety-like behaviors, while P2X7R knock-out mice show reduced anxiety. P2X7R antagonists can reduce anxiety and depression-like behaviors in a mouse model of chronic social stress. Combining P2X7R antagonists with commonly used antidepressants can improve antidepressant efficacy, and thus may lead to new antidepressant treatment strategies [31]. As mentioned, the P2X7R and associated inflammasomes are involved in stress-induced neuroinflammation, providing further motivation for the search for objective biomarkers and therapeutic targets for depression.

## Role of the P2X7R in anxiety or depression in individuals with comorbid somatic disease

Chronic diseases are the leading cause of death worldwide. In the adults with somatic disease, the rate of co-occuring symptoms of anxiety and depression is higher than 20%, which increases their morbidity and mortality [32]. The surface expression of P2X7R on peripheral blood mononuclear cells in patients with primary Sjogren’s syndrome (pSS) was significantly higher than that in a control group, suggesting that the P2X7R may modulate the complex pathogenesis of pSS as well as symptoms of anxiety and/or depression [33]. Mice undergoing oophorectomy experience a decrease in estrogen levels, leading to an increase in P2X7R expression, and IL-1β,IL-18,NLRP3 inflammasome activation leads to hippocampal neuroinflammation and depression [34].

Chronic pain is frequently comorbid with anxiety and/or depression. The incidence of comorbid anxiety or depression is significantly higher in populations with chronic pain than that in normal populations. Currently, information about the comorbidity mechanism between chronic pain and secondary anxiety/depression is relatively limited. ATP appears to activate the P2X7R, which opens non-selective cation channels, activates multiple intracellular signaling pathways, and releases multiple inflammatory cytokines, causing nerve damage and exacerbating pain [35]. The P2X7R is a trigger for inflammatory factors, and is widely distributed in tumor cells and various immune cells. By releasing pro-inflammatory cytokines, it mediates inflammatory responses and can regulate tumor proliferation, chronic pain, and central nervous system inflammation [36]. Studies have indicated that purinergic inflammation reactions in microglia cells and changes in neuronal plasticity in the hippocampal CA1 on the same side may be key pathogenic mechanisms underlying trigeminal neuralgia-induced anxiety/depression. Trigeminal neuralgia activates microglia in the ipsilateral hippocampus of mice through ATP/P2X7 receptor interaction, This leads to the upregulation of ATP on the same side of the Hippocampus, along with IL-1 β. Long-term potentiation (LTP) is impaired in the hippocampus following the induction of depressive-like behaviors in rodents [37]. In summary, the relationships among pain, anxiety, and depression are complex. Thus, there may be interactions and influences among them. Pain may trigger anxiety and depression, which can also exacerbate pain perception and experience. Further research is needed to explore whether this relationship is bidirectional.

The activation of P2X7 receptors can trigger a series of intracellular signaling cascade reactions, leading to the release of inflammatory mediators, such as cytokines and chemokines. These inflammatory mediators can further affect the excitability of neurons and changes in neurotransmitters, thereby promoting the occurrence of epilepsy and its comorbid, anxiety and depression. Therefore, by regulating the activity of P2X7 receptors, treating epilepsy and comorbid anxiety and depression simultaneously is possible [38].

## P2X7R in comorbid anxiety and depression: the inflammation hypothesis

**Fig. 1:**
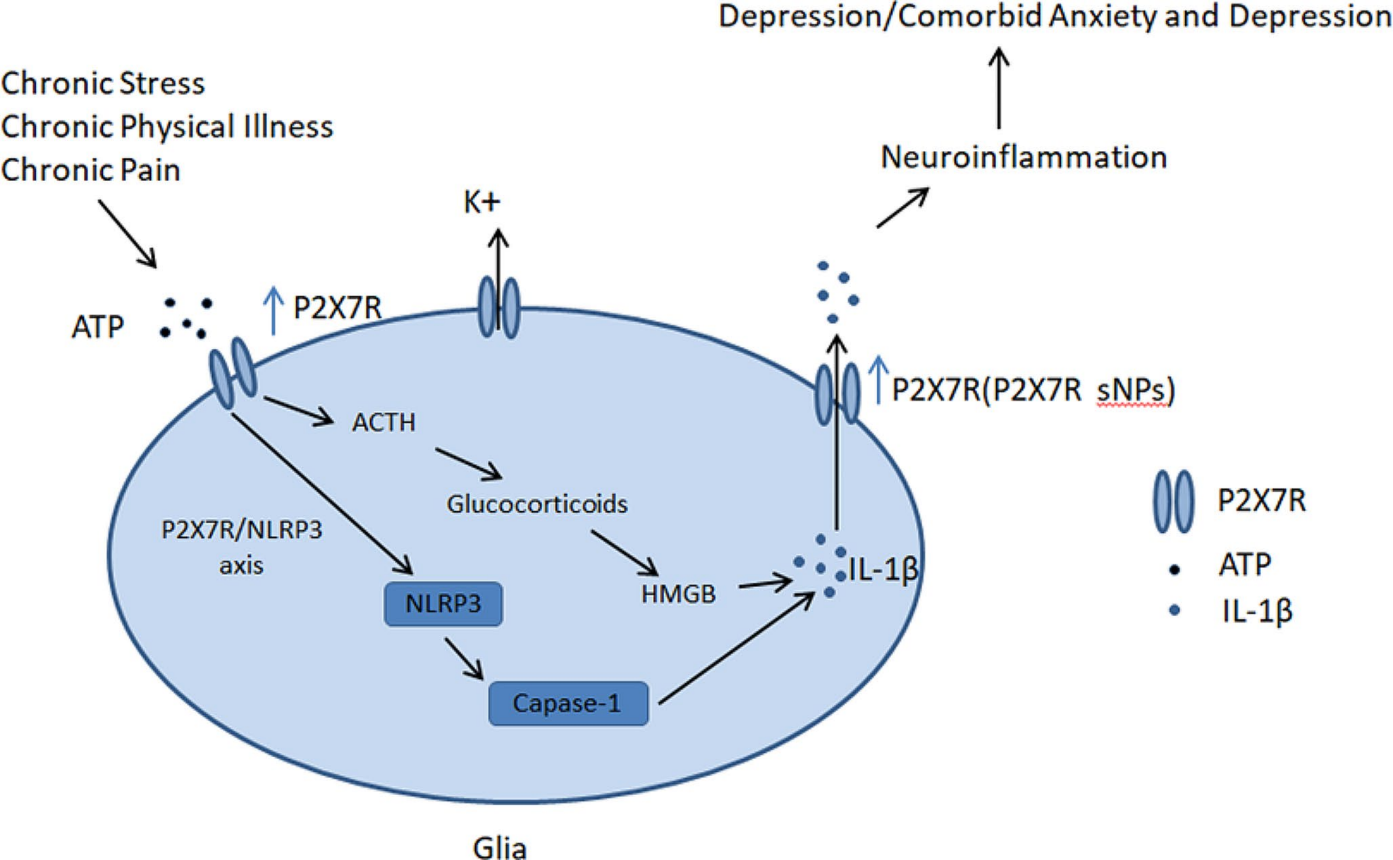
The mechanism of P2X7R mediated glia activation and comorbid anxiety and depression. Chronic stress, pain, or physical illnesses increase extracellular ATP, serving as a stimulus signal to activate P2X7R on glial cells. Once activated, P2X7R triggers ACTH secretion, enhancing the release of HMGB and IL-1β. Additionally, P2X7R mediates K + efflux and activates caspase-1 via NLRP3, promoting IL-1β conversion. Prolonged ATP-induced activation of P2X7R also forms a larger pore channel, facilitating the release of mature IL-1β, further exacerbating neuroinflammation and comorbid anxiety and depression

Although many studies using animal models, behavioral tests, cell experiments, and pharmacological manipulations have examined the relationship between the P2X7R and depression, few have considered the involvement of the P2X7R in anxiety disorders. As mentioned above, the P2X7R may be involved, to varying degrees, in the development of depression and anxiety, with differential P2X7R gene expression in individuals with anxiety and depression. Animal experiments have shown that increased P2X7R expression can lead to depression-like behavior that can be alleviated via P2X7R antagonists and P2X7R knockouts. Clinical studies have also found that in some patients with somatic diseases and comorbid anxiety and depression, increased P2X7R expression triggers neuroinflammatory responses, leading to anxiety and depression (Fig. 1). We hypothesize that the P2X7R may be an ideal therapeutic target for comorbid anxiety and depression; however, further clinical research is needed to confirm this hypothesis.

## The potential diagnostic value of the P2X7R

### P2X7R SNPs and susceptibility to anxiety and depression

P2X7R is highly polymorphic, and when it is in the central nervous system, it acts as an innate immune cell that mediates neuroinflammation. It is also involved in the activation and expression of certain transcription factors and plays a crucial regulatory role in inflammation at the gene transcription level. Inhibiting P2X7 channel activation can reduce microglial cell activation and inflammation, indicating that P2X7R mitigates neuronal death and neurodegenerative processes. Consequently, various drugs have been developed to target the P2X7 channel [39]. Polymorphisms are widely present in the human P2X7 receptor gene, and these contribute to changes in amino acid sequences that can alter receptor function and possibly influence susceptibility to various neuropsychiatric disorders [40]. As P2X7R gene variations have different effects on anxiety and depression, distinct biological mechanisms may be involved in the treatment of anxiety and depression via targeting P2X7Rs [41]. Multiple studies have shown that P2X7Rs promote neuroinflammation, which can lead to the development of mood disorders. For instance, a specific P2X7R SNP (rs67881993) was found to influence neuroinflammation, and childhood trauma exacerbated this inflammatory response, thereby intensifying symptoms of anxiety disorders [42]. A genotype analysis in humans revealed a relationship between P2X7R polymorphisms and anxiety disorders. Certain variations in the P2X7R gene may increase the risk of anxiety disorders, while some variations in the CaMKKb gene may modulate anxiety disorder severity [43].

Results from animal studies have shown that mice expressing the human P2X7R and carrying the Gln460Arg variant are more prone to depression under conditions of chronic stress. Compared with Gln460 allele carriers, Arg460 allele carriers have poorer sleep quality and experience more instances of awakening. Furthermore, the Arg460 allele is associated with changes in P2X7 receptor function, which could lead to imbalances in specific neurotransmitters and reduced emotional processing abilities. Therefore, the Gln460Arg polymorphism of the P2X7 gene could serve as a biomarker for predicting the risk of depression and sleep disorders and provide a basis for personalized treatment [44].

A cross-sectional study showed that the Gln460Arg polymorphism, which is an SNP in the P2X7R gene, can lead to functional changes in the P2X7R. This polymorphism has two alleles: Gln and Arg. The Gln460Arg polymorphism of the P2RX7 gene is associated with the severity of depression symptoms. In other words, individuals who carry the Arg/Arg genotype are more likely to experience severe depressive symptoms than those with the Gln/Gln genotype. These findings contribute to our understanding of the pathogenesis of depression and could provide new avenues for personalized depression treatment. However, further confirmation of these findings in larger patient populations is needed [45]. A case-control analysis was conducted on 315 hospitalized patients with acute severe depression and 406 healthy control subjects. Based on these findings, the P2RX7 haplotype combination of Gln460Arg and adjacent SNPs contributes to the observed genetic association with depressive symptoms [46]. All of these studies demonstrate that P2X7R gene polymorphisms could provide individualized diagnosis and treatment options for individuals with depression, anxiety disorder, or both.

The expression of P2X7R has been investigated in the context of anxiety and depression, showing a potential link between P2X7R expression levels and the severity of these conditions. Xie et al. found that surface expression of P2X7R on peripheral blood mononuclear cells (PBMC) in patients with primary Sjögren’s syndrome (pSS) was significantly higher than in controls and suggested that P2X7R may contribute to the complex pathogenesis of pSS as well as anxiety and/or depression [33]. This study also found that P2X7R expression on CD14- PBMC was significantly positively correlated to scores of anxiety and depression, indicating a potential parallel between P2X7R expression levels and the severity of these mood disorders. Another study focusing on genetic polymorphisms associated with the P2X7 receptor found that certain polymorphisms were linked to the severity of depressive symptoms in patients with mood disorders, suggesting a genetic basis for the observed variations in P2X7R expression and its impact on mood disorders [47]. These findings suggest a complex interaction between P2X7R expression, genetic predisposition, and the pathophysiology of anxiety and depression.

### Using PET imaging to monitor P2X7R expression

Under normal circumstances, the expression of P2X7R may be relatively stable but may change during inflammation or neurotransmitter disorders. Positron emission tomography (PET) imaging is gradually being applied to the evaluation of psychiatric disorders that involve neuroinflammation, including depression, anxiety disorders, and schizophrenia [48].Recent research has focused on 18 kDa translocator protein imaging and radioligands that target other inflammatory markers [49, 50].

The P2X7R represents a novel molecular target for imaging neuroinflammation using PET. GSK1482160, a potent P2X7R antagonist, exhibits high receptor affinity, excellent BBB penetration, and the capacity for radiolabeling with 11 C. It can specifically bind to the P2X7R in vivo, allowing for real-time, non-invasive visualization of the extent and location of neuroinflammation in the brain using PET imaging. Compared with other radiotracers, 11 C-GSK1482160 offers higher specificity and sensitivity. Thus, 11 C-GSK1482160 is a promising radioligand that could be used to target the P2X7R, enabling it to serve as a biomarker of neuroinflammation [51]. This radiotracer could facilitate the diagnosis and treatment of neuroinflammation while enhancing our understanding of the mechanisms underlying neuroinflammation and potential therapeutic approaches.

Since the P2X7R promotes neuroinflammation, which leads to symptoms of anxiety and depression, measuring P2X7R expression via PET imaging could facilitate the clinical diagnosis and treatment of patients with comorbid anxiety and depression.

## The mechanism of action of antidepressants

Antidepressants in clinical practice include rapid antidepressants and typical antidepressants. Both directly bind to the transmembrane domain of the tyrosine kinase receptor 2 (TRKB), thereby promoting synaptic localization of the TRKB and the activation of the brain-driven neurogenic factor. Rapid antidepressants (ADs), such as ketamine (KET), can easily penetrate the brain to quickly reach a sufficient synaptic concentration. The concentration of typical AD in the brain gradually increases to the level required for TRKB binding and takes a long time to take effect [52]. KET, a fast-acting AD, may change clinical treatment, but due to its short duration and hallucinogenic nature, its clinical application is limited, and other drugs with similar AD effects must be developed [53].

Classic antidepressant drugs in clinical practice mainly include Selective Serotonin Reuptake Inhibitors**(**SSRIs) and Selective Norepinephrine Reuptake Inhibitors(SNRIs), which increase the concentration of these neurotransmitters in the synaptic gap by inhibiting the reuptake of serotonin and norepinephrine, thereby regulating neurotransmission. They can also have anti-inflammatory effects, improve emotions, and reduce anxiety by inhibiting the production of pro-inflammatory cytokines, reducing oxidative stress and regulating the activity of immune cells [54]. However, these antidepressants usually take several weeks before their therapeutic effects manifest; thus, developing antidepressants that can quickly take effect is an important research direction. Precise treatment drugs based on the different types and severity of anxiety and depression and tailored to individual characteristics are a current research trend. These may include personalized drug selection based on genotype, phenotype, or other biomarkers [55, 56].

## Potential therapeutic value of the P2X7R

P2X7R, as a key participant in initiating the inflammatory signaling cascade, has become a potential target for antidepressant drug research [57]. Some animal experiments have shown that P2X7R antagonists may exert rapid anti-anxiety and depression effects by reducing neuroinflammation and regulating the neurotransmitter system.

### Potential therapeutic targets for comorbid anxiety and depression

During stress, extracellular ATP increases in the hippocampus, leading to increased cytokine release and activation of the P2X7 receptor-mediated NLRP3 inflammasome. This results in an increase in depressive-like behavior. Mice lacking P2X7Rs do not exhibit anxiety and depressive-like behavior. Furthermore, P2X7 receptor agonists can induce depressive-like behavior in mice, while P2X7R inhibitors can prevent anxiety and depressive-like behavior induced by chronic unpredictable stress in rats. Therefore, the P2X7/NLRP3 axis may be a potential therapeutic target for stress-induced psychiatric disorders [28].A study using a humanized microglia-specific in vitro model demonstrated that the human P2X7R is activated in response to stress and immune stimuli, and that this activation leads to an increase in levels of pro-inflammatory cytokines such as IL-1β, IL-6, and TNF-α. Human P2X7R antagonists can reduce pro-inflammatory cytokine levels and increase IL-4 secretion. These findings may contribute to our understanding of the role of the P2X7R in mediating neuroinflammation in psychiatric disorders, and facilitate the development of new treatment strategies [58]. In a mouse model of chronic mild stress (UCMS), activated microglia secreted inflammatory factors. Thus, P2X7 receptor antagonists might reduce the activation state of microglia and lower the secretion of inflammatory factors such as IL-1β and TNF-α. The UCMS model led to a neuroendocrine imbalance, with increased CRH and AVP secretion. In this model, P2X7 receptor antagonists could be used to regulate hormone secretion and improve neuroendocrine abnormalities. Previous research suggests that P2X7 receptor antagonists could improve behavioral symptoms, microglial activation, and neuroendocrine abnormalities in individuals with depression, thus providing new targets for novel treatments for depression [59].

Psychological stress increases the release of ATP in microglial cells and stimulates the expression of purinergic P2X7Rs. This promotes activation of the NLRP3 inflammasome in the hippocampus, leading to the release of the inflammatory cytokine IL-1β. Simultaneously, inflammasome activation impairs synaptic plasticity, affecting neuronal connectivity and signaling. P2X7R antagonists can block the release of IL-1β and TNF-α, while activating NLRP3. Psychological stress is signaled via the ATP/P2X7R-NLRP3 inflammasome cascade response, which has led to new therapeutic targets for treating stress-related mood disorders and identifying comorbidities [27].

In some acute animal models of severe depression, such as the tail suspension and forced swim tests, microinjections of the P2X7R agonist ATP or its analog, 2’,3’-O-(2,4,6-trinitrophenyl)-ATP (TNP-ATP) into the medial prefrontal cortex can exacerbate depressive-like behavior in mice. These effects can be reversed by the P2X7R antagonist JNJ-47,965,567 [60].Chronic stress and Hypothalamic-Pituitary-Adrenal(HPA) axis overactivation may trigger a cascade reaction in the serotonin system. Studies have shown that inhibition of the P2X7 receptor with Brilliant Blue G can improve chronic stress-induced activation of microglial cells in the cerebral cortex, hippocampus, and basal ganglia of mice, restoring balance to the HPA axis [59]. In a contextual fear conditioning memory study in mice, P2X7Rs mediated neuronal activity during the consolidation of fear memories by regulating intracellular calcium levels. During the extinction of fear memories, P2X7Rs mediated neuronal plasticity, allowing for the reprogramming of new neurons and thereby erasing old fear memories. In terms of memory expression, P2X7R activation can facilitate the phosphorylation of cAMP-response element binding protein and c-fos proto-oncogene protein, promoting neuronal activity and memory expression. In summary, P2X7Rs play a crucial role in the expression and extinction of fear memories. Selective P2X7R antagonists impair the extinction learning of contextual fear memories, thereby influencing anxiety behavior. This suggests that the P2X7R may have differing roles in depression and anxiety [61]. A carborane cage (4 and Cs·5) exerts a significant biological effect as an inhibitor of the purinergic P2X7 receptor (P2X7R), which allows for targeting depression in vivo and thus demonstrates, for the first time, that a carborane has the capacity to modify CNS activity [62]. A P2X7 antagonist that can penetrate the central nervous system is under development as an imaging tracer for brain inflammation and as an antidepressant treatment drug [41].

### Targets for treating comorbid anxiety and depression in individuals with somatic diseases

Following nerve injuries, P2X7Rs can bind to ATP, which activates and attracts immune cells into pain-related areas and triggers an inflammatory response that amplifies pain signals. The use of broad-spectrum P2X7 antagonists, such as Pyridoxal-5-phosphate(PPADS) and TNP-ATP, can inhibit P2X7R activity, reducing the release of pro-inflammatory factors and mitigating both the inflammatory response and tissue damage. This suggests that the P2X7R may play a crucial role in comorbid chronic pain with anxiety or depression [63]. Animal model experiments have discovered that gallic acid, also known as 3,4,5-trihydroxybenzoic acid, inhibits ferroptosis in spinal cord microglial cells and alleviates behavioral changes in rats with comorbid pain and depression by modulating the P2X7-ROS signaling pathway [64].

Treatment of ovariectomized (OVX) mice with inflammasome inhibitor VX-765 can improve depressive and anxiety-like behavior, and reverse IL-1 in the hippocampus β elevated levels of IL-18. Estrogen receptor agonists can also reverse P2X7R expression, regulate hippocampal inflammation in female mice, and improve depression- and anxiety-like behavior [34]. A knockdown of the ipsilateral hippocampal P2X7R of mice prevented trigeminal neuralgia-induced microglial activation and anxiodepressive-like behaviors. Thus, targeting signal transduction between microglia cells and P2X7Rs may provide new strategies for treating chronic pain with comorbid anxiety and depression [37].

Individuals with epilepsy, and especially those with drug-resistant epilepsy, are at a greater risk of comorbid depression or anxiety. Conventional antiepileptic drug treatments often have suboptimal outcomes and are associated with a range of side effects. Given that P2X7R antagonists can alleviate neuroinflammatory responses and neuronal damage, modulating P2X7R activity may enable the suppression of epileptic seizures and protect against comorbidities such as anxiety and depression [38].

## Conclusion and future prospects

1) Increasing evidence suggests that the P2X7R may be a valuable therapeutic target for anxiety, depression, and comorbid conditions. Modulating the P2X7R may influence neurotransmitter release and synaptic plasticity, alleviate neuroinflammation, and reduce oxidative stress, thus modulating the neural circuits associated with anxiety and depression. However, further research is needed to validate the effectiveness and safety of P2X7Rs as a treatment target, as well as to explore combined use with other treatment methods.

2) Genetic polymorphisms of the P2X7R are associated with different types of psychiatric disorders, although current research is limited. P2X7R expression varies in different animal models of anxiety and depression, as well as in patients with comorbid anxiety and depression of different etiologies. There is a potential parallel between P2X7R expression levels and the severity of these mood disorders. Further exploration of variations in P2X7R expression and mechanisms according to symptom type, severity levels, and comorbidities in animal models and patients with anxiety and depression could provide new insights regarding the diagnosis and treatment of these conditions.

3) Changes in P2X7R expression are not specific to anxiety and depression, and alterations in purinergic signaling alone cannot serve as independent diagnostic criteria. PET imaging holds significant potential in the field of neuroinflammation and is gradually being applied to assess neuroinflammation in psychiatric disorders. Utilizing PET imaging to evaluate P2X7R expression in the brain as a measure of the extent and location of neuroinflammation may offer new avenues for the clinical diagnosis and treatment of patients with anxiety and depression.

4) P2X7R inhibitors can suppress glial cell activation and control neuroinflammation by penetrating the BBB to target microglial cells and P2X7R signaling. Thus, they may provide new strategies for the treatment of anxiety, depression, and their comorbidities. However, more clinical research is needed. Specifically, future studies in which P2X7R inhibitors are combined with antidepressant medications could help to determine more precise antidepressant treatment strategies, especially in patients with comorbid anxiety and depression.

## Data Availability

No datasets were generated or analysed during the current study.
